# Supplementary material for: Oxygen/glucose-deprivation causes long-term impairment of synaptic CaMKII movement
Source: bioRxiv. 2025 Mar 4:2025.03.01.640973. Preprint. [Version 1] doi: 10.1101/2025.03.01.640973 (PMC11908153; doi:10.1101/2025.03.01.640973)
Supplement: Supplement 1 [file NIHPP2025.03.01.640973v1-supplement-1.pdf]

bioRxiv preprint doi: https://doi.org/10.1101/2025.03.01.640973; this version posted March 4, 2025. The copyright holder for this preprint (which was not certified by peer review) is the author/funder, who has granted bioRxiv a license to display the preprint in perpetuity. It is made available under aCC-BY 4.0 International license.

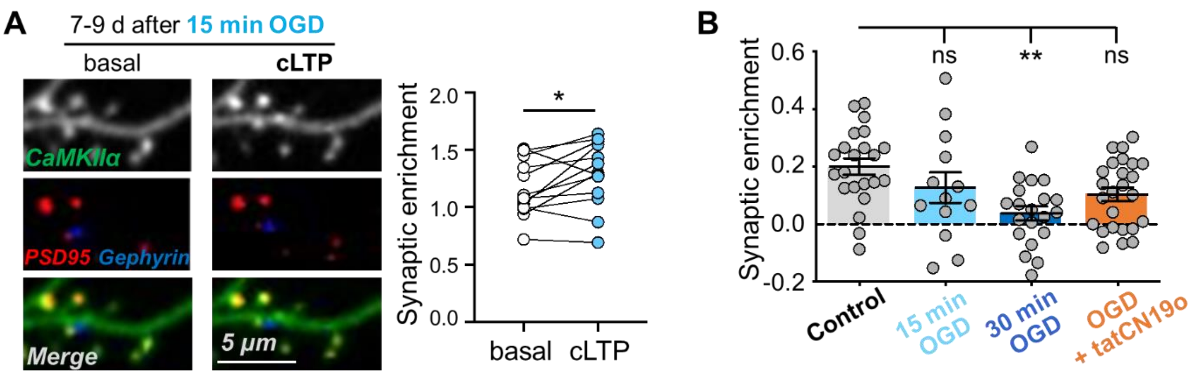

Supplemental Figure S1: 15 min of OGD has no significant effect on CaMKII movement.
(A) In contrast to 30 min OGD (see Fig. 1B), 15 min OGD does not completely block cLTP-induced CaMKII movement to excitatory synapses on day 7-9 after the insult (paired, two-tailed t-test, *p=0.0322, n=13 cells).
(B) Bar graph quantification shows the relative change in basal vs. cLTP after control (grey), 15 min OGD (light blue), 30 min OGD (dark blue), or 30 min OGD plus tatCN19o (orange) treatment. The change in synaptic enrichment of CaMKII is significantly lower after 30 min OGD, compared to control (one-way ANOVA with Tukey post-hoc test; control vs. 15 min OGD p=0.3422; control vs. 30 min OGD **p=0.0020; control vs. 30 min OGD + tatCN19o ns p=0.1217). Error bars indicate s.e.m.
